# Association of hybridization and genome doubling with codon usage landscapes in the subgenomes of bread wheat

**DOI:** 10.3389/fpls.2026.1857428

**Published:** 2026-09-07

**Authors:** Xingchen Li, Mengyao Li, Yongchao Hao, Chengyu Liu, Yuhang Gao, Conghui Jiang, Xueqiang Wang, Muhammad Abdul Rehman Rashid, Yan Zhao

**Affiliations:** 1 State Key Laboratory of Wheat Improvement, College of Agronomy, Shandong Agricultural University, Tai’an, China; 2 State Key Laboratory of Wheat Improvement, Peking University Institute of Advanced Agricultural Sciences, Shandong Laboratory of Advanced Agricultural Sciences, Weifang, China; 3 College of Resources and Environment, Shandong Agricultural University, Tai’an, China; 4 Institute of Wetland Agriculture and Ecology, Shandong Academy of Agricultural Sciences, Jinan, China; 5 Hainan Yazhou Bay Seed Laboratory, Sanya, Hainan, China; 6 Department of Bioinformatics and Biotechnology, Government College University Faisalabad, Faisalabad, Pakistan

**Keywords:** allopolyploidization, asymmetric subgenome changes, codon usage, gene structure, wheat

## Abstract

**Introduction:**

The success of allopolyploids is partly attributable to their ability to resolve genomic conflicts during the early generations following allopolyploidization. Codon usage patterns represent important genomic signatures, and codon usage bias can influence gene expression and cellular function through multiple processes. However, little is known about how subgenomic conflicts in codon usage are resolved during allopolyploidization.

**Methods:**

We characterized changes in codon usage from the wild progenitors of bread wheat to modern bread wheat. In addition, we generated full-length transcriptomes of *Aegilops tauschii* (male parent), *Triticum durum* (female parent), their triploid hybrids, and spontaneously doubled allohexaploid wheat to investigate changes in codon usage associated with hybridization and genome doubling.

**Results:**

The evolutionarily divergent subgenomes of bread wheat exhibited similar codon usage patterns, whereas the codon usage patterns of bread wheat differed markedly from those of its wild progenitors, which tended to have higher GC content at the third codon position (GC3). These differences were associated with asymmetric changes in subgenomic GC3 during the transition from the wild progenitors to bread wheat. Changes in GC3 were also associated with sharp decreases in the average number of transcripts from GC3-poor genes. Analysis of the synthetic allopolyploidization system further revealed distinct changes in codon usage during successive stages of bread wheat allopolyploidization, including hybridization and genome doubling. In particular, genome doubling was accompanied by an increase in GC3, contributing to the GC3-rich codon usage bias observed in bread wheat.

**Discussion:**

These findings reveal dynamic remodeling and convergence of codon usage patterns during wheat allopolyploidization and suggest that hybridization and genome doubling contribute differently to this process. Our results provide new insights into the evolution of codon usage landscapes in allopolyploids and may improve our understanding of subgenomic accommodation following allopolyploidization.

## Introduction

Bread wheat (*Triticum aestivum* L., AABBDD) was formed approximately 10,000 years ago by hybridization between cultivated tetraploid emmer wheat (*Triticum turgidum* L., AABB) and wild diploid *Aegilops tauschii* (DD), followed by chromosome doubling, a process known as allopolyploidy (Mallet, 2007; Mayer et al., 2014; Zhou et al., 2020). Rapid and diverse transcriptomic responses to allopolyploidization have been documented in bread wheat and other allopolyploid systems (Chang et al., 2010; Wang et al., 2006; Alix et al., 2017; Yu et al., 2020; Li et al., 2021; Glombik et al., 2021; Qin et al., 2021; Ramírez-González et al., 2018), and these responses indicate that the structure of the genome is highly plastic_ENREF_4. An increasing number of studies have shown that these induced transcriptomic changes in allopolyploids might help to overcome subgenomic incompatibilities, which promotes environmental adaptation in early generations (Leitch and Leitch, 2008; Doyle et al., 2008). However, much remains to be learned regarding how allopolyploidization-induced transcriptomic responses may contribute to genome stabilization following the allapolyplloydization.

Codon usage varies substantially across the tree of life (Muyle et al., 2011; Mitreva et al., 2006; Hodgman et al., 2020; Southworth et al., 2018; Galtier et al., 2018; Novoa et al., 2019). In bread wheat, a relatively young species, patterns of codon bias differ from those of its wild progenitors at the whole-genome level ^21^. Mass transcriptomic changes related to codon usage have been reported in bread wheat, including changes in gene structure, alternative splicing, and gene loss (Yu et al., 2020; Qin et al., 2021; Ramírez-González et al., 2018; Yang et al., 2021; Akhunov et al., 2013). Although previous studies have demonstrated that various factors might interfere with codon usage, the origin and evolutionary pressures driving patterns of codon bias remain largely unknown.

The total GC content of coding sequences (GC-CDS), rather than the local GC of genomic sequences (GC-GS), is correlated with cross-species differences in the frequencies of codons (Muto and Osawa, 1987; Osawa et al., 1988). GC-CDS was shown to be the strongest determinant of codon usage variation across species through an analysis of a large dataset from various taxa (311 bacteria, 28 archaea, and 257 eukaryotes) (Knight et al., 2001). The GC content in the third codon site (GC3) is more strongly correlated with codon usage compared with the GC content in the first and second codon sites (GC1 and GC2, respectively) (Schwartz et al., 2009; Figuet et al., 2014). GC-CDS and GC-GS have been recently described in various species (Serres-Giardi et al., 2012; Lemaire et al., 2019; Bohlin and Pettersson, 2019; Iyer, 2012), and these properties can be used as genomic signatures to distinguish species (Serres-Giardi et al., 2012; Bohlin and Pettersson, 2019; Pracana et al., 2020; Hamilton et al., 2016; Alföldi et al., 2011). In angiosperms (but not in mammals), GC-CDS is not or at most weakly correlated with local GC-GS, which is attributed to the highly dynamic nature of angiosperm genomes, including greater frequencies of polyploidy and greater levels of recombination (Kejnovsky et al., 2009). However, little is known about the extent to which GC-CDS landscapes are affected by allopolyploidization-induced subgenomic changes.

Three non-mutually exclusive hypotheses have been proposed to explain GC landscape evolution: selection on codon usage in coding regions, mutational bias, and GC-biased gene conversion (Southworth et al., 2018; Galtier et al., 2018; Serres-Giardi et al., 2012; Hamilton et al., 2016; Behura and Severson, 2013; Long et al., 2018). GC-biased gene conversion might play a significant role in the evolution of GC-CDS in plant genomes (Serres-Giardi et al., 2012). However, the relative contributions of these three hypotheses to shaping patterns of GC-CDS are not realized until after several generations, suggesting that their effects are independent of allopolyploidization-induced genome changes in early generations. A unifying hypothesis proposed for angiosperm genomes (particularly those of grasses) posits that gene structure can affect GC-CDS patterns along with recombination patterns and GC-biased gene conversion (Glémin et al., 2014). However, gene structure is complex and affected by several variables. Consequently, one of the problems associated with determining the causes of observed GC-CDS change during bread wheat allopolyploidization is the difficulty of untangling its causes and consequences given that allopolyploidization can affect several genic variables.

In this study, we report the codon usage landscapes of bread wheat and its wild progenitors and identify the genomic features associated with a codon usage during the bread wheat allopolyploidization. Evolutionarily divergent genomes in bread wheat share the same codon usage pattern of GC3-rich codons, which might be directly induced by asymmetric increases in GC-CDS from wild progenitors to bread wheat, especially GC3 increases. Estimations of the contributions of multiple gene structure variables to GC3 increases revealed that GC3 increases were strongly associated with decreases in the average number of splice variants, suggesting that transcript structural changes may contribute to GC3 evolution during allopolyploidization. Variation in codon usage and GC3 among evolutionarily divergent genomes in bread wheat can be explained by hybridization and genome doubling. GC3 increases are observed during genome doubling, which results in a GC3-rich codon bias within bread wheat.

## Results

### Evolutionarily divergent subgenomes in bread wheat have the same codon usage patterns

**Figure 1:**
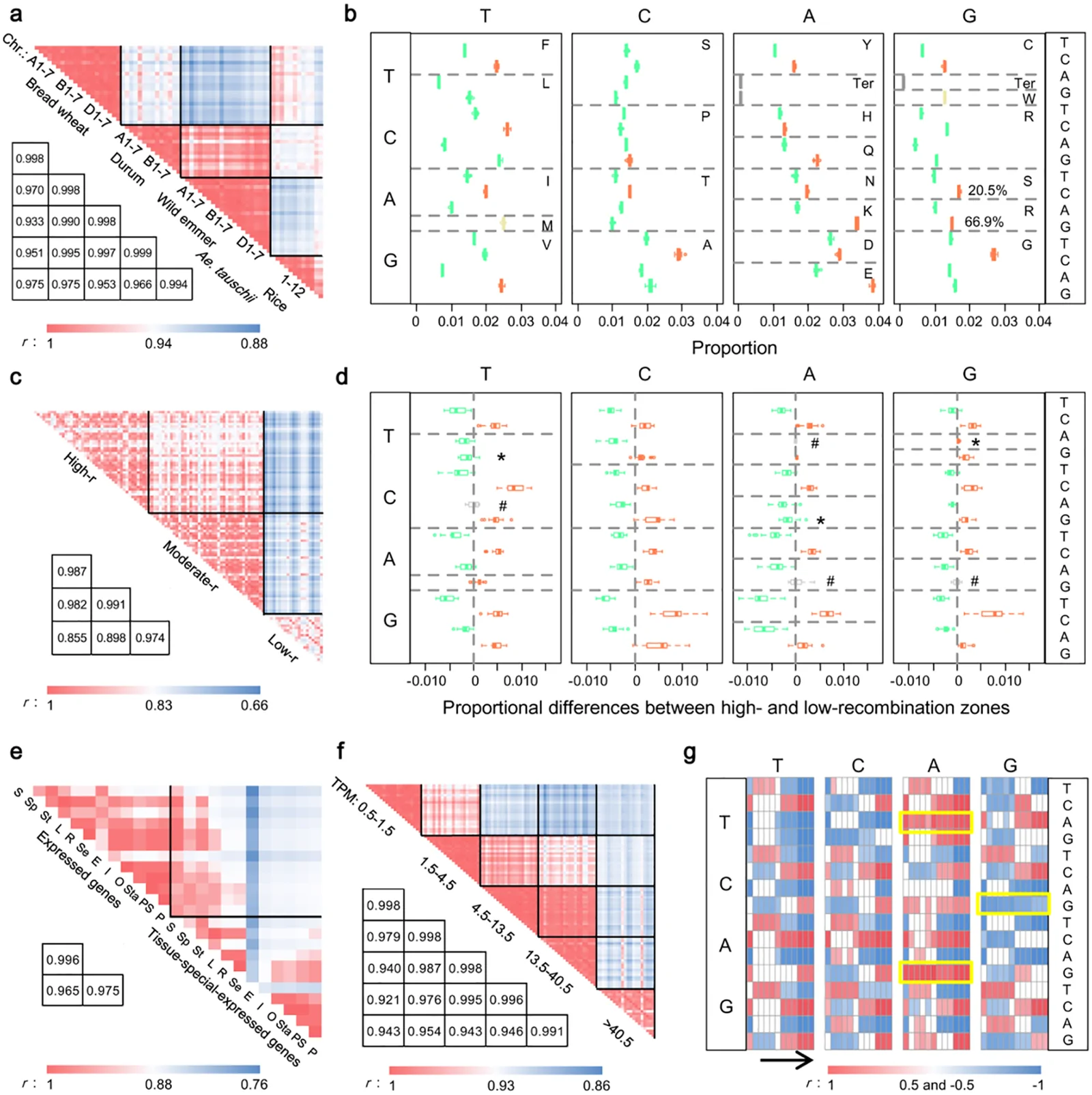
Changes in codon usage from wild progenitors to bread wheat. **(a)** A heatmap depicting pairwise correlations in chromosomal codon composition across bread wheat, durum, wild emmer, *Ae. tauschii*, and rice. Chromosome abbreviations of all species are in parentheses. The numbers in the lower triangle are the mean correlation coefficients in chromosomal codon composition between corresponding species. **(b)** Codon composition of bread wheat at the chromosome level. The y-axis shows proportions. According to the function or corresponding amino acid type of the codon, the boxes of 64 codons are plotted together and separated by dashed lines. The three termination codons are shown in gray, and tryptophan (W) and methionine (M) residues are shown in yellow. Orange and green indicate the highest frequency codon accounting for 18 amino acids and the other codons, respectively. **(c)** A heatmap depicting pairwise correlations in codon composition within differential recombination zones of bread wheat, including 42 high-, 42 moderate-, and 21 low-recombination zones. The abbreviations of high-, moderate-, and low-recombination zones are in parentheses. The numbers in the lower triangle show the mean correlation coefficients in codon composition for the corresponding differential recombination zones. **(d)** Differences in codon composition between high- and low-recombination zones in bread wheat. Colored boxes show significant differences in the proportions of codons, and gray boxes show the others. Orange indicates higher proportions in the high-recombination zones than in the low-recombination zones for the corresponding codon (28 codons with G or C in the third codon site and TGA), whereas blue indicates lower proportions in the high-recombination zones than in the low-recombination zones for the corresponding codon (29 codons with A or T in the third codon site, and TTG and CAG). TGA, TTG, and CAG are marked by *. The symbol # indicates no statistically significant difference between the high- and low-recombination zones. **(e)** Correlations in codon composition using all expressed genes and genes showing tissue-specific expression within seed (S), spike (Sp), stem (St), leaf (L), root (R), seeding (Se), endosperm (E), inner pericarp (I), outer pericarp (O), stamen (Sta), pistil stamen (PS), and pistil (P). Tissue acronyms are in parentheses. The numbers in the lower triangle show the mean correlation coefficients in codon composition for all expressed genes and genes showing tissue-specific expression within 12 bread wheat tissues. **(f)** Correlations in codon composition among five differential TPM groups: 0.5 to 1.5, 1.5 to 4.5, 4.5 to 13.5, 13.5 to 40.5, and more than 40.5. **(g)** Correlations between the proportion of each codon and gene expression level within 12 bread wheat tissues. Within each codon plot, the 12 tissues are placed along the direction of the arrow, including S, Sp, St, L, R, Se, E, I, O, Sta, PS, and (P) White represents no correlation (-0.5< r 0.5). Three codons that may affect gene expression are marked with yellow borders.

To explore patterns of codon usage within bread wheat, all high-confidence protein-coding sequences (CDSs) of bread wheat (Chinese Spring) IWGSC RefSeq annotation V1.0, accession GCA-900519105.1 (Appels et al., 2018) were obtained; CDSs from i) durum (Svevo) genome assembly, V1.0/V1.1, accession GCA-900231445.1 (Maccaferri et al., 2019), ii) wild emmer (Zavitan), WEWSeq gene annotation v1.0, accession GCA-002162155.1 (Avni et al., 2017), iii) *Ae*. *tauschii* (AL8/78), HCC gene set v2.0, Aet v4.0, GCA-002575655.1 (Luo et al., 2017), and iv) rice (Nipponbare) IRGSP map-based sequence/IRGSP Build 5, accessions AP008207–AP008218 (for 12 chromosomes) were used as controls (Materials and Methods). For each chromosome of each of the above species, the proportions of 64 codons were integrated into a vector showing the codon composition of the corresponding chromosome (Materials and Methods). Nearly perfect correlations in codon composition were detected between any two bread wheat chromosomes (Mean *r*: 0.998), which were higher than those between bread wheat and the other four species (Mean *r*: 0.933 to 0.975) (**Figure 1A**). Levels of variation were moderate among the 21 bread wheat chromosomes (0.93% to 3.68%) compared with the chromosomes of the other four species (**Supplementary Figure S1**). Generally, there was no significant difference in the proportion of each codon between the AB and D subgenome in bread wheat (**Supplementary Figure S2**; **Supplementary Table 1**), and the 18 highest frequency codons accounting for 18 amino acids [proportion among their corresponding synonymous codons: 20.5% (AGC) to 66.9% (AAG)] all had G or C at the third codon site (**Figure 1B**).

To characterize the composition of codons along bread wheat chromosomes, the codon composition was determined in 10-Mb sliding windows with a step size of 5 Mb (Materials and Methods). Strong correlations were detected among all sliding windows of each chromosome, except for windows around the pericentromeric region (**Supplementary Figure S3**). After splitting bread wheat chromosomes into 42 high-, 42 moderate-, and 21 low-recombination zones (Materials and Methods) (Appels et al., 2018), relatively low correlations were detected between low- and high-recombination zones (Mean *r*: 0.855) and between low- and moderate-combination zones (Mean *r*: 0.898); the correlation between high- and moderate-recombination zones was high (Mean *r*: 0.987) (**Figure 1C**; **Supplementary Figure S3**). High- and moderate-recombination zones had a significantly higher proportion of codons with G or C at the third codon site (28 and 27) and a significantly lower proportion of codons with A or T at the third codon site (29 and 31) compared with low-recombination zones (**Figure 1D**; **Supplementary Figures S4, S5**). Strong correlations in codon composition were detected among 42 high- (Mean *r*: 0.987), 42 moderate- (Mean *r*: 0.991), and 21 low-recombination zones (Mean *r*: 0.974), respectively (**Figure 1C**). For each of the three sets of differential recombination zones above, there was no significant difference in the proportion of each codon between the AB and D subgenome in bread wheat (**Supplementary Table 1**).

### Bread wheat subgenomes have the same codon usage at the tissue level

To explore the codon usage of different tissues, we extracted expressed genes and genes showing tissue-specific expression for 12 bread wheat tissues (including seed, spike, stem, leaf, root, seedling, endosperm, inner pericarp, outer pericarp, stamen, pistillody stamen, and pistil) (Materials and Methods). Strong correlations in codon composition were detected among the 21 bread wheat chromosomes for 12 tissues based on the set of expressed genes (Mean *r*: 0.997 to 0.999) and genes showing tissue-specific expression (Mean *r*: 0.986 to 0.998) (**Supplementary Table 2**). Few (ATA in endosperm, ACG and GTT in root, and ACG in stamen) and no significant differences were observed between the AB and D subgenome for each codon based on the set of expressed genes and genes showing tissue-specific expression (**Supplementary Table 3**). Among the 12 bread wheat tissues, we also detected strong correlations in codon composition using the set of expressed genes (Mean *r*: 0.996) and genes showing tissue-specific expression (Mean *r*: 0.975) (**Figure 1e**).

To examine the relationship between gene expression level and codon composition, we divided the expressed genes into five groups according to transcripts per million (TPM) (Materials and Methods). For all expressed genes across the 12 bread wheat tissues, codon-composition correlations gradually declined from 0.998 to 0.921 with increasing TPM differences (**Figure 1f**). The pattern above remained when the five differential TPM groups within each tissue were used (**Supplementary Figure S6**), and most codons showed positive (14 to 26 codons, *r* > 0.5) or negative (17 to 31 codons, *r*< -0.5) correlations between their proportions and gene expression levels (**Figure 1g**). Finally, negative (r< -0.5) and positive (r > 0.5) correlations were consistently observed between the proportion of a codon and the gene expression level for one (CGG) and two (AAG and TAA) common codons within each of the 12 bread wheat tissues (**Figure 1g**), suggesting that these three codons are associated with gene expression levels. Whether these codons directly influence transcriptional or translational regulations remains to be determined. Nevertheless, for each differential TPM group within each tissue, strong correlations in codon composition were observed among the 21 chromosomes (Mean *r*: 0.852 to 0.997) (**Supplementary Figure S6**), and no significant difference in the proportion of each codon was observed between the bread wheat AB and D subgenome (**Supplementary Table 4**).

### Bread wheat tends to have more GC3-rich codons than its wild progenitors

**Figure 2:**
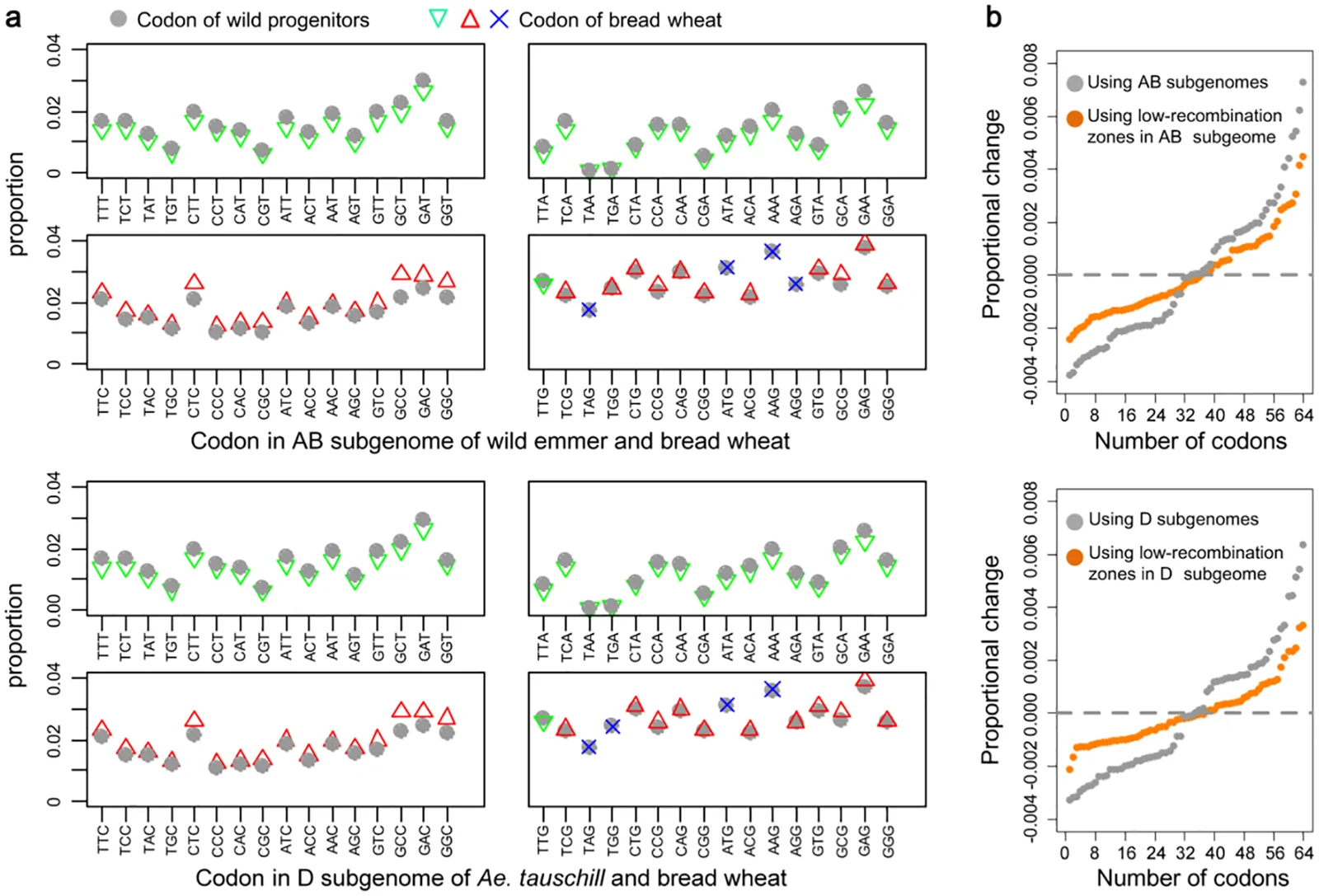
Changes in codon composition from wild progenitors to bread wheat. **(a)** The proportions of 64 codons within bread wheat and its wild progenitors in the AB (top) and D subgenomes (bottom). Gray dots indicate the codons of wild progenitors, and the others show the codons of bread wheat. Red and green triangles indicate significant increases and decreases in the proportions of codons from wild progenitors to bread wheat. Blue crosses indicate no significant change from wild progenitors to bread wheat. **(b)** Changes in the proportion of each codon from wild progenitors to bread wheat in the AB (top) and D (bottom) subgenomes and low-recombination zones within. The codons are arranged in ascending order according to the magnitudes of changes in their proportions.

We investigated the characteristics of bread wheat codon usage at the chromosomal level and compared them with those of its wild progenitors (*T. dicoccoides* and *Ae. tauschii*). Significant differences (*p* < 0.05) in the proportion of nearly all codons (AB: 60 and D: 60) were observed between bread wheat and its wild progenitors in the AB (except ATG, AGG, TAG, and AAG) and D subgenome (except ATG, AGG, TAG, and TGG) (**Figure 2a**; **Supplementary Figure S7**). Changes in the proportions of all codons ranged from -0.38% to 0.73% and from -0.33% to 0.64% in the AB and D subgenome, respectively (**Figure 2b**; **Supplementary Figure S8**). Significant decreases were observed in the proportions of 33 common codons in the AB and D subgenome from wild progenitors to bread wheat; 32 of these were codons with A or T at the third codon site. Significant increases in the proportion of codons (AB: 28 and D: 28) with G or C at the third codon site were observed (**Figure 2a**; **Supplementary Figure S8**). Generally, bread wheat tends to have more GC3-rich codons than its wild progenitors at the chromosome level.

To determine whether recombination affects changes in codon composition, we characterized changes in codon composition along chromosomes from wild progenitors to bread wheat. The number of significant changes in the proportions of codons observed was fewer (AB: 49 and D: 20) from wild progenitors to bread wheat and the range of variation was smaller (AB: -0.24% to 0.45% and D: -0.21% to 0.33%) in low-recombination zones relative to that at the level of entire chromosomes (**Figures 2b**; **Supplementary Figures S9, S10**). For the AB subgenome, A or T was observed at the third codon site in 28 of the 29 codons showing significant decreases (*p* < 0.05) in their proportions from wild progenitors to bread wheat, and C or G was observed at the third codon site for all 20 codons showing significant increases in their proportions from wild progenitors to bread wheat (**Supplementary Figure S10**). For the D subgenome, A or T was observed at the third codon site for 11 of 12 codons showing significant decreases in their proportions from wild progenitors to bread wheat, and G or C was observed at the third codon site for all eight codons showing significant increases in their proportions from wild progenitors to bread wheat (**Supplementary Figure S10**). These findings indicate that the low-recombination zones of bread wheat also tended to have more GC3-rich codons compared with their wild progenitors, although the magnitude of the change in codon composition from wild progenitors to bread wheat was lower when low-recombination zones were used than when whole chromosomes were used.

### Bread wheat codon usage patterns might contribute to the asymmetry in the increase in GC3 from wild progenitors to bread wheat

**Figure 3:**
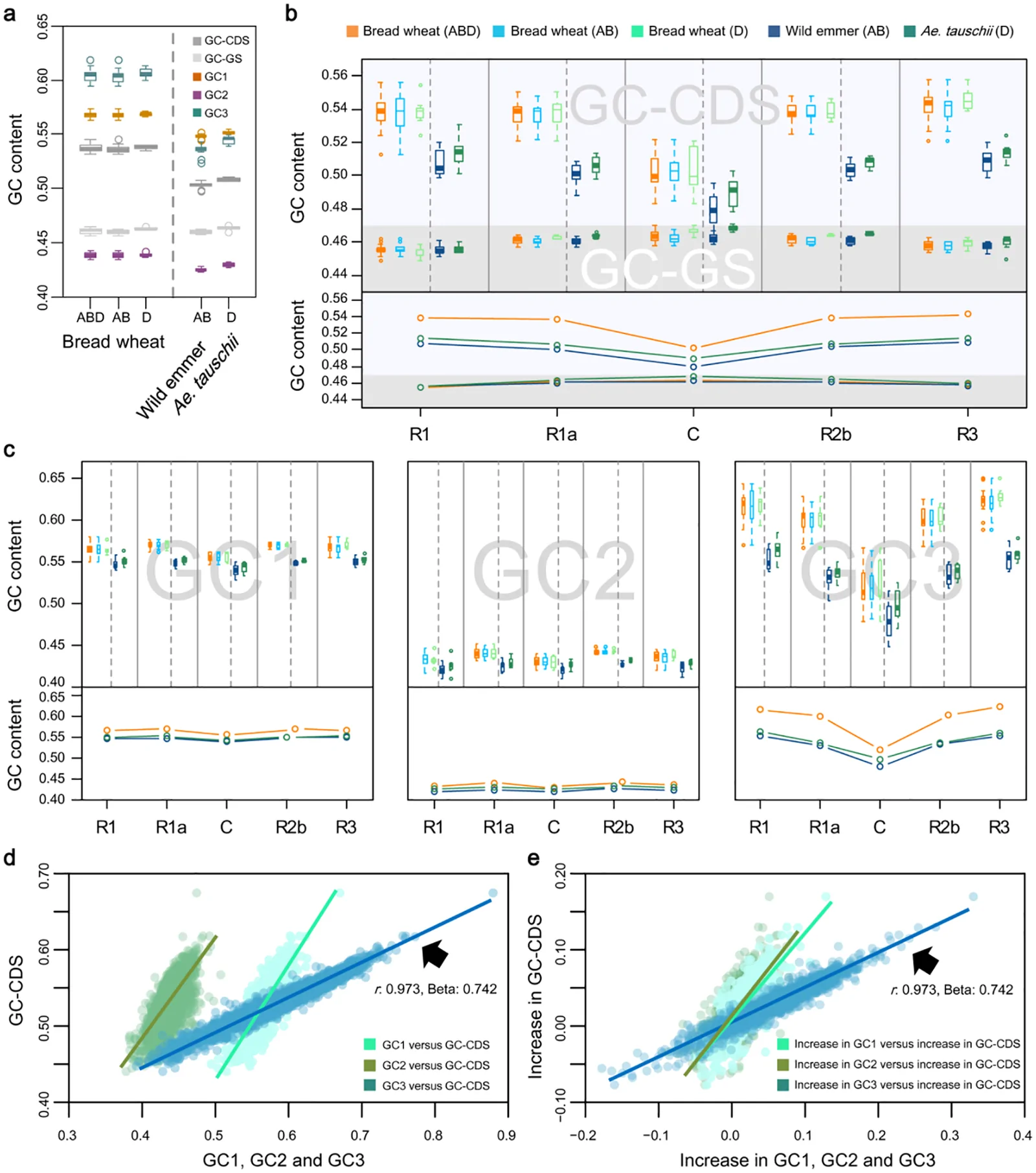
Changes in the GC content from wild progenitors to bread wheat. **(a)** GC-CDS, GC1, GC2, GC3, and GC-GS of bread wheat and its wild progenitors at the chromosome level. The boxes of bread wheat, wild emmer, and *Ae. tauschii* are plotted together and separated by dashed lines. Gray boxes show GC-CDS (dark gray) and GC-GS (light gray), and colored boxes indicate GC1 (orange), GC2 (purple), and GC3 (blue). **(b)** Box (top) and line (bottom) charts on GC-CDS (light background) and GC-GS (deep background) within five differential recombination zones within bread wheat and its wild progenitors at the chromosomal window level. The y-axis shows the five differential recombination zones. **(c)** Box (top) and line (bottom) charts on GC1 (left), GC2 (middle), and GC3 (right) of five differential recombination zones within bread wheat and its wild progenitors at the chromosomal window level. **(d)** Relationships of GC-CDS with GC1, GC2, and GC3 at the chromosomal window level. **(e)** Relationship among increases in GC-CDS with increases in GC1, GC2, and GC3 from wild progenitors to bread wheat at the chromosomal window level.

We investigated changes in GC-CDS from wild progenitors to bread wheat; GC-CDS is considered a direct indicator of codon usage (Novoa et al., 2019; Knight et al., 2001; Palidwor et al., 2010). First, no significant differences (*p<* 0.05) in GC-CDS were observed between the AB and D subgenomes of bread wheat (53.66% and 53.81%, respectively), which was significantly higher than that of wild emmer (AB: 50.31%) and *Ae. tauschii* (D: 50.86%) (**Figure 3a**; **Supplementary Figure S11**). Low-recombination zones had significantly lower GC-CDS than the other zones within bread wheat (AB: 50.17% and D: 50.39%), wild emmer (AB: 48.00%), and *Ae. tauschii* (D: 48.98%) (**Figure 3b**; **Supplementary Figure S12**). A significant asymmetry in the increase in subgenome GC-CDS was observed (AB: 3.35% versus D: 2.95%) from wild progenitors to bread wheat, even within low-recombination zones (AB: 2.17% versus D: 1.41%) (**Figure 3b**; **Supplementary Figure S13**).

We also investigated the contributions to the increase in GC-CDS from each of the three codon sites (referred to as GC1, GC2, and GC3). As expected, there was no significant difference between the bread wheat AB and D subgenome in GC1 (56.77% and 56.86%, respectively), GC2 (43.84% and 43.90%, respectively), and GC3 (60.37% and 60.65%, respectively) (**Figures 3a, c**; **Supplementary Figure S14**). GC3 might be the main determiner of the chromosomal window GC-CDS in bread wheat with correlation coefficients and standardized partial regression coefficients of 0.973 and 0.742, respectively (**Figure 3d**). GC1, GC2, and GC3 were significantly lower in wild emmer (54.82%, 42.51%, and 53.60%, respectively) and *Ae. tauschii* (55.18%, 42.98%, and 54.40%, respectively) than in bread wheat (**Figures 3a, c**; **Supplementary Figures S14, S15**). For low-recombination zones, GC1, GC2, and GC3 were lower along the chromosomes of bread wheat (AB: 55.53%, 43.01%, and 51.89, respectively) (D: 55.64%, 43.01%, and 52.52, respectively), wild emmer (AB: 53.96%, 42.06%, and 47.98%, respectively) and *Ae. tauschii* (D: 55.27%, 42.72%, and 49.86%, respectively) (**Figure 3c**; **Supplementary Figure S16**). Significant asymmetries in increases in subgenome GC1, GC2, and GC3 from wild progenitors to bread wheat were also observed (AB: 1.96%, 1.33%, and 6.78% versus D: 1.68%, 0.92%, and 6.25%, respectively) (**Figures 3a, c**; **Supplementary Figure S17**). The increase in GC3 might be the main determiner of the increase in GC-CDS, as the correlation coefficient and the standardized partial regression coefficients were 0.952 and 0.692, respectively (**Figure 3e**).

We tested the effect of the GC-GS landscape on the formation of the GC-CDS landscape in bread wheat. GC-CDS was significantly higher than GC-GS (53.71% versus 46.09%) in bread wheat (**Figure 3a**; **Supplementary Figures S11, S12**); no correlations or negative correlations between GC-CDS and its local GC-GS were detected for the 21 chromosomes (*r*: -0.48 to 0.15) (**Supplementary Figure S18**). A previous study has shown that GC-GS is significantly lower in bread wheat than its wild progenitors in corresponding subgenomes (Zhao et al., 2020). There was no correlation or a weak correlation (*r*: -0.38 to 0.31) between increases in GC-CDS and local GC-GS changes from wild progenitors to bread wheat (**Supplementary Figure S19**). The results suggested that GC-CDS and increases in GC-CDS were less dependent on local GC-GS and GC-GS change from wild progenitors to bread wheat.

### Increases in GC3 are strongly associated with reduction in transcript number, particularly among GC3-poor genes from wild progenitors to bread wheat

**Figure 4:**
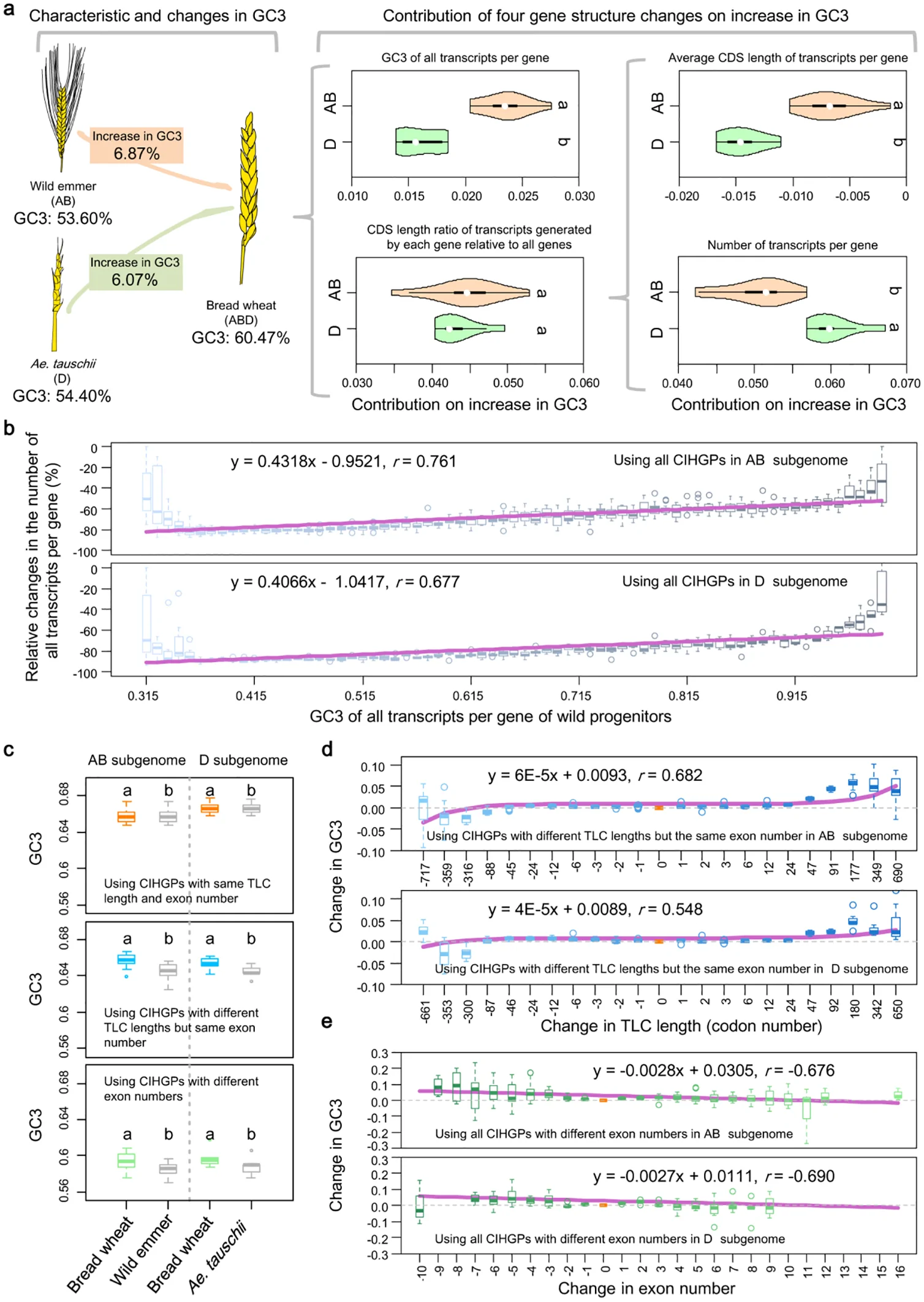
Changes in gene structure and their contribution to increases in GC3 from wild progenitors to bread wheat. **(a)** Characteristics and changes in GC3 from wild progenitors to bread wheat (left) and the contribution of these changes to increases in GC3 of four gene structure variables (right): GC3 of all transcripts per gene, CDS length ratio of transcripts generated by each gene relative to all genes, average CDS length of transcripts per gene, and number of transcripts per gene. For violins within each plot, adjacent letters indicate significant differences (*p* < 0.05) according to an independent-sample t-test. **(b)** Correlation between GC3 of all transcripts per gene of wild progenitors and relative changes in the number of all transcripts per gene within its corresponding CIHGP in the AB (top) and D subgenome (bottom). **(c)** GC3 of bread wheat and wild progenitors using CIHGPs with the same TLC length and exon number (top), CIHGPs with different TLC lengths but the same exon number (middle), and CIHGPs with different exon numbers (bottom). For boxes within each plot, letters indicate significant differences (*p* < 0.05) according to paired *t*-tests. **(d)** Correlations between changes in GC3 and changes in TLC length within CIHGPs with different TLC lengths but the same exon number in the AB (top) and D (bottom) subgenome. **(e)** Correlations between changes in GC3 and changes in exon number within CIHGPs with different exon numbers in the AB (top) and D subgenome (bottom).

The GC3 in a species can be denoted as $\text{GC}3=\sum x_{i}(n_{i}/N)$, where *x_i_* and *n_i_* represent GC3 and the length of the CDS of *i*th transcript, respectively, and N is the total CDS length of all transcripts. According to the above formula, increases in GC3 involved two main changes in gene structure: GC3 of all transcripts per gene and the CDS length ratio of transcripts generated by each gene relative to all genes. The CDS length ratio of transcripts generated by each gene to all genes was determined by measuring two variables: number of transcripts per gene and the average CDS length of transcripts per gene. Using the collinearity-incorporating homologous gene pairs (CIHGP) within bread wheat and its wild progenitors (Chen et al., 2020), we estimated the effect of the above four forms of structural variation on increases in GC3 (Materials and Methods). Changes in the GC3 of all transcripts per gene made moderate contributions to increases in GC3 from wild progenitors to bread wheat (AB: 2.36% and D: 1.60%), and changes in the CDS length ratio of transcripts generated by each gene to all genes might be the main contributor to increases in GC3 (AB: 4.40% and D: 4.35%) (**Figure 4a**). Changes in the number of transcripts per gene made the greatest contributions to increases in GC3 (AB: 5.00% and D: 6.03%), whereas changes in the average CDS length of transcripts per gene made a negative contribution to increases in GC3 (AB: -0.65% and D: -1.43%) (**Figure 4a**). The contributions of three distinct changes in gene structure (GC3 of all transcripts per gene, average CDS length of transcripts per gene, and number of transcripts per gene) to increases in GC3 significantly differed between the AB and D subgenome (**Figure 4a**).

To characterize changes in the number of transcripts and average CDS length of transcripts per gene from wild progenitors to bread wheat, we divided the CIHGPs of each chromosome into 100 groups based on the GC3 of all transcripts per gene of the wild progenitors (**Figure 4b**). Among all CIHGP groups in the AB and D subgenomes, there were significant decreases in the number of transcripts per gene (AB: -34.20% to -81.93% and D: -20.00% to -89.57%) (**Figure 4b**; **Supplementary Figure S20**) and increases in the average CDS length of transcripts per gene (AB: 24.42% to 73.45% and D: 4.92% to 59.09%) from wild progenitors to bread wheat (**Supplementary Figures S21, S22**). GC3-poor genes of wild progenitors tended to lose more transcripts per gene in the AB (*r*: 0.761) and D (*r*: 0.667) subgenome from wild progenitors to bread wheat (**Figure 4b**) and have higher average CDS lengths of transcripts per gene in the AB (*r*: -0.447) and D (*r*: -0.594) subgenome (**Supplementary Figure S21**). These results suggest that reduction in transcript number, particularly in GC3-poor genes, are strongly associated with increases in GC3 and may contribute to the observed evolutionary pattern from wild progenitors to bread wheat. However, additional functional studies will be required to establish the direct causal relationships.

Transcripts with the longest CDS (TLC) per gene were used to study the effect of changes in other genic structures on increases in GC3 from wild progenitors to bread wheat. The GC3 of TLC was significantly larger than that of un-TLC in bread wheat (67.67% versus 54.69%), wild emmer (65.42% versus 54.33%), and *Ae. tauschii* (64.60% versus 55.56%), suggesting that a GC3-poor exon bias plays a role in mediating changes from TLC to un-TLC within bread wheat and its wild progenitors (**Supplementary Figure S23**). A significant asymmetry in increases in subgenome GC3 was observed in the TLC of CIHGPs (AB: 0.86% versus D: 0.99%) (**Supplementary Figures S24, S25**). Based on differences in exon number and TLC length, we divided the CIHGPs into three groups including CIHGPs with the same TLC length and exon number, CIHGPs with different TLC lengths but the same exon number, and CIHGPs with different exon numbers (**Supplementary Figure S26**). Significant and larger increases in GC3 were observed for the TLC of CIHGPs with different TLC lengths but the same exon numbers (AB: 1.11% and D: 0.90%) and CIHGPs with different exon numbers (AB: 0.88% and D: 0.92%) compared with CIHGPs with the same TLC length and exon number (AB: 0.03% and D: 0.03%) (**Figure 4c**). Further correlation analysis showed that increases in TLC length were positively associated with increases in GC3 in the AB (*r*: 0.682) and D (*r*: 0.548) subgenome from wild progenitors to bread wheat (**Figure 4d**), whereas increases in exon number had opposite effects in the AB (*r*: -0.676) and D (*r*: -0.690) subgenome (**Figure 4e**).

### Increases in GC3 are associated with genome doubling following hybridization during allopolyploidization in bread wheat

**Figure 5:**
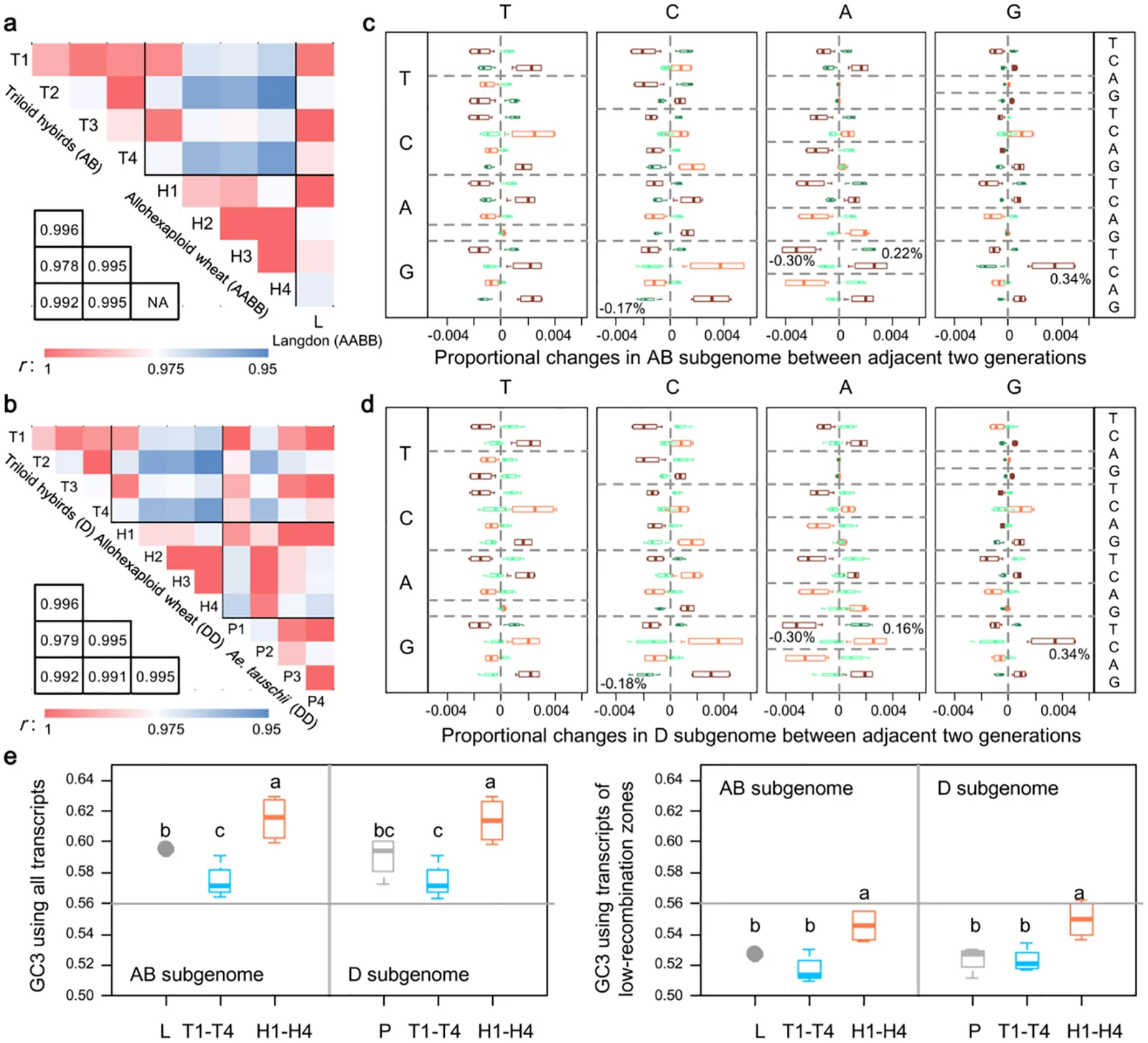
Changes in codon composition in bread wheat allopolyploidization, involving hybridization and genome doubling. **(a)** A heatmap depicting pairwise correlations in codon composition in the AB subgenome of four triploid hybrids (T1 to T4), four doubled wheat allohexaploids (H1 to H4), and Langdon **(L)**. Parent-child three-generation quartets are in parentheses. The numbers in the lower triangle indicate the mean correlation coefficients in codon composition for the corresponding generation. **(b)** A heatmap depicting pairwise correlations in codon composition in the D subgenome of four triploid hybrids (T1 to T4), four doubled wheat allohexaploids (H1 to H4), and *Ae. tauschii* (P1 to P4). **(c)** Changes in the proportions of each codon in the AB subgenome between Langdon and triploid hybrids (green) and between triploid hybrids and doubled allohexaploid wheat (red). Dark green and red boxes indicate significant changes in the proportions of codons according to paired *t*-tests. The numbers above boxes are extreme values of changes in proportions during hybridization and genome doubling. **(d)** Changes in the proportion of each codon in the D subgenome between *Ae. tauschii* and triploid hybrids (green), as well as those between triploid hybrids and doubled allohexaploid wheat (red). **(e)** GC3 using all transcripts (left) and GC3 using transcripts of low-recombination zones (right) within Langdon (L), triploid hybrids (T1-T4), and doubled allohexaploid wheat (H1-H4) in the AB subgenome and within *Ae. tauschii* (P), triploid hybrids, and doubled allohexaploid wheat in the D subgenome. Gray dots show the corresponding values of one Langdon, and the colored box shows values from four triploid hybrids and four doubled allohexaploid wheat. For boxes within each plot, letters indicate significant differences (*p* < 0.05) according to Duncan’s test.

To test the effects of hybridization and genome doubling on changes in the composition of codons during allopolyploidization in bread wheat, parent-child three-generation quartet isoform sequencing was performed by PacBio Iso-Seq using four groups of interspecific crosses [four *Ae. tauschii* (DD), one common tetraploid wheat (AABB), four triploid hybrids (ABD), and four doubled wheat allohexaploids (AABBDD)] (Materials and Methods). Correlations in the composition of codons between the AB and D subgenome were nearly perfect (*r* ≈ 1) within each of four triploid hybrids and four doubled wheat allohexaploids (**Supplementary Figure S27**), and correlations in the composition of codons between the low-recombination zones and the other zones were low (**Supplementary Figure S28**). The correlation in the composition of codons between triploid hybrids and doubled allohexaploid wheat was lowest in the AB and D subgenome (Mean *r*: 0.978 and 0.979, respectively) compared with that between triploid hybrids and parents (Mean *r*: 0.992 and 0.992, respectively) and that between doubled allohexaploid wheat and parents (Mean *r*: 0.995 and 0.991, respectively) (**Figures 5a, b**). These findings indicate that changes in the composition of codons from triploid hybrids to the doubled allohexaploids are larger than those from parents to triploid hybrids.

Significant changes in the proportions of codons were fewer (AB: 37 and D: 16) and the range of variation was smaller [AB: -0.17% (GCG) to 0.22% (GAT) and D: -0.18% (GCG) to 0.16% (GAT)] during hybridization than during genome doubling (AB: 40 and D: 35) [AB: -0.30% (GAT) to 0.34% (GGC) and D: -0.30% (GAT) and 0.33% (GGC)] (**Figures 5c, d**; **Supplementary Figures S29, S30**). Significant decreases on the basis of paired *t*-tests in the proportion of 22 and 11 codons in the AB and D subgenome during hybridization were observed, and 19 and 9 of these codons had G or C at the third codon site, respectively. Significant increases (*p* < 0.05) in the proportions of 15 and five codons in the AB and D subgenome were observed during hybridization, and 14 and five of these codons had an A or T at the third codon site, respectively (**Figures 5c, d**). By contrast, significant increases in the proportions of 19 and 16 codons in the AB and D subgenome were observed during genome doubling, and G or C was present in the third codon site in 19 and 16 of these codons, respectively. Significant decreases in the proportions of 21 and 19 codons in the AB and D subgenome were observed during genome doubling, and A or T was present at the third codon site of 20 and 18 of these codons, respectively (**Figures 5c, d**). For low-recombination zones, significant changes in the proportions of codons were also fewer (AB: 26 and D: 11) and the range of variation in the proportions of codons was also smaller [AB: -0.11% (TTC) to 0.20% (GAT) and D: -0.08% (CCA) to 0.08% (GGT)] during hybridization, compared with during genome doubling (AB: 29 and D: 26) [AB: -0.20% (GAT) to 0.24% (GGC) and D: -0.20% (GAT) and 0.25% (GGC)] (**Supplementary Figure S31**). Significant decreases in the proportions of 15 and eight codons were observed in the AB and D subgenome during hybridization, and G or C was present in the third codon site of 11 and three of these codons, respectively. Significant decreases in the proportions of 11 and three codons in the AB and D subgenome were observed during hybridization, and A or T was present at the third codon site in eight and two of these codons, respectively (**Supplementary Figure S31**). Significant increases in the proportions of 12 and 11 codons in the AB and D subgenomes were observed during genome doubling, and G or C was present in the third codon site of 12 and 11 of these codons, respectively. Significant decreases in the proportions of 17 and 15 codons in the AB and D subgenomes were observed during genome doubling, and A or T was present in the third codon site of 17 and 14 of these codons, respectively (**Supplementary Figure S31**). Overall, GC3-rich codon bias was most pronounced in doubled allohexaploid wheat, followed by the parents; the weakest GC3-rich codon bias was observed in triploid hybrids (**Figures 5c, d**; **Supplementary Figure S31**).

GC3, the underlying determinant of codon usage, decreased during hybridization (AB: -2.04% and D: -1.61%), and this was accompanied by a decrease in GC-CDS (AB: -0.89% and D: -0.86%) (**Figure 5e**; **Supplementary Figure S32**). GC3 then increased significantly during genome doubling (AB: 4.05% and D: 3.95%), and this was accompanied by a significant increase in GC-CDS (AB: 1.70%, D: 1.66%) (**Figure 5e**; **Supplementary Figure S32**). Significant asymmetries in increases in subgenome GC3 and GC-CDS were observed during genome doubling (**Supplementary Figure S33**). For low-recombination zones, there was a very weak decrease in GC3 (AB: -1.06% and D: -0.09%) and GC-CDS (AB: -0.50% and D: -0.30%) during hybridization. Furthermore, significant increases in GC3 (AB: 2.87% and D: 2.65%) and GC-CDS (AB: 1.18% and D: 1.16%) were observed in low-recombination zones during genome doubling (**Figure 5e**; **Supplementary Figure S34**). Overall, GC3 decreased during hybridization transition and increased during the subsequent genome-doubling across the genome, resulting in GC3-poor codon usage in triploid hybrids and GC3-rich codon usage in doubled allohexaploid wheat.

## Discussion

The understanding of why subgenomes have changed following hybridization and genome doubling remains limited (Leitch and Leitch, 2008; Doyle et al., 2008; Qiu et al., 2020; Chen, 2007; Janko et al., 2021). Here, we aimed to address this question from a novel perspective by surveying the codon usage landscapes in bread wheat and the underlying mechanisms shaping them. The results of our study revealed patterns of codon usage in different parts of the genome and transcriptome, including between evolutionarily divergent subgenomes, between low-recombination zones and other recombination zones, among regions with different expression levels, and among different tissues. Our findings also provide new insights into the relationships among a few critical components in genome/subgenome evolution during allopolyploidization: “codon usage”, “GC-GS”, “GC-CDS”, “gene structure”, “hybridization”, and “genome doubling”. Our findings highlight associations among codon composition and GC-CDS, gene structure, hybridization, and genome doubling during bread wheat allopolyploidization. Differences in codon usage and GC-CDS between low-recombination zones and the other-recombination zones were observed in triploid hybrids and doubled allohexaploid wheat. Because these patterns were detectable in the experimentally generated material, they are consistent with the possibility that their establishment does not require long-term evolutionary divergence alone; however, the effects of mutation, selection, and GC-biased gene conversion cannot be excluded. In addition, we demonstrated the efficacy of our backward reasoning strategy for clarifying relationships between allopolyploidization-induced transcriptomic responses associated with genome stabilization after allopolyploidization.

In this study, there were some consistent correlations between the increase in GC3 content, codon-usage changes and transcript structural variations during the process of bread wheat allopolyploidization; however, there are several alternative biological and technical explanations for these patterns. The first is that some wheat genome assemblies and annotations are of higher quality than the ones available for the progenitors, which may lead to variations in the estimation of coding sequence composition, number of transcripts, and diversity of isoforms. The identification of transcripts is very sensitive to the quality of the annotations, as alternative splicing and transcript reconstruction still are not easy even using long-read sequencing technologies (Tardaguila et al., 2018). To reduce these possible biases, we included comparative analysis with publicly available reference genomes along with PacBio Iso-Seq data produced from new synthetic triploid hybrids and double allohexaploid wheat, which were then analyzed using a common analytical framework. However, some of the variation observed may be due to differences in the releases of annotations or the recovery of the transcript. While the accuracy of transcript annotations is significantly improved by full-length Iso-Seq, these differences in annotation, sequencing depth and/or transcript recovery cannot be ruled out as factors affecting the estimate of transcript number. Thus, it is important to view these observed decreases in the number of transcripts as transcriptomic associations, and not definitive biological loss of isoforms. Additionally, as the publicly available reference genomes belonged to different accessions, some differences observed may be due to genetic variation between accessions. The regular trends seen in independently synthesized allopolyploids of known parental lines, however, indicate that the larger trends reported here are not likely to be due to the differences in the accessions themselves.

An important consideration is that differences in transcript number among the compared genomes may reflect not only the changes in transcript structure or isoform composition but also the differences in gene copy number and presence-absence variation. The analyses in this study was based on annotated transcript models and collinearity-incorporating homolgous gene pairs, hence, didn’t independently quantify the copy-number variation or genome-wide presence-absence variation for each material. Resultantly, the estimated contribution of transcript-number genes to GC3 variation should be interpreted as an association with changes in annotated transcript composition. Therefore, any future study incorporating high-quality genome assemblies and explicit copy-number and presence-absence variation assessment may required to disentangle these effects.

There are several biological processes that could affect the evolution of GC3 after allopolyploidization aside from the technical factors. Polyploidy in plants has been reported to drive expression bias among homoeologs, differential gene loss versus retention, selection of expressed genes based on their level of expression, and expression-dependent alternative splicing (Adams and Wendel, 2005; Chen, 2007; Doyle et al., 2008). The evolution of codon usage is a result of many forces, such as mutational bias, natural selection for translational efficiency and accuracy, and GC-biased gene conversion (Plotkin and Kudla, 2011; Glémin et al., 2014). In addition, when genes are highly expressed, they often display higher codon bias due to the selection on translation efficiency and fidelity (Hershberg and Petrov, 2008; Plotkin and Kudla, 2011). These findings mean that the codon preference of bread wheat for GC3 is probably a result of multiple mechanisms that occurred during the merger of the genomes, genome doubling, long-term evolutionary processes and transcriptome remodeling, and not just one. The correlation seen in both natural bread wheat and independently synthesized allohexaploids suggests a link between genome doubling and increases in GC3, but the results presented here should be viewed as providing candidate mechanisms and not direct causation. Combination of matched genome sequences, quantitative analyses of simultaneous expression of homoeologs, ribosome-profiling, tRNA abundance, and functional validation would add value to the study in the future to distinguish the relative importance of these processes in codon-usage evolution upon allopolyploidization.

In angiosperms, allopolyploidy triggers a new steady state for the transcriptome, metabolome, and proteome networks and might enhance network robustness and/or increase network complexity (Leitch and Leitch, 2008; Doyle et al., 2008; Qiu et al., 2020; Chen, 2007). Genomic plasticity enables changes in the original steady state, which equips newly originated polyploid individuals with novel biochemical pathways and transgressive characters that allow them to exploit new niches and/or outcompete diploid parents (Leitch and Leitch, 2008; Doyle et al., 2008). Harnessing the genomic plasticity of bread wheat to create excellent germplasm resources is necessary for increasing the genetic diversity of nascent allopolyploids. Our comparison and synthesis allopolyploid analyses have found consistent associations of transcript structure with the GC3 increases observed in allopolyploid plants, but have not been able to completely untangle the effects of allopolyploidization, domestication, and subsequent evolutionary processes. So the observed correlations do not necessarily imply a causal relationship. An improved understanding of genomic plasticity would enhance our ability to develop high-quality wheat germplasms through artificial synthesis and distant hybridization (Glémin et al., 2019). Although much has been learned regarding bread wheat allopolyploidization over the last decade (Glémin et al., 2019), many outstanding issues remain, such as the evolutionary force that drives genomic changes during hybridization and doubling, how various subgenomic conflicts are resolved, and the origin of novel phenotypes and environmental adaptation. Comprehensive genomic data on *Triticum* species and innovations in computing algorithms will help resolve these issues and aid improvements in the quality of wheat germplasm.

## Materials and methods

### Characterizing changes in codon composition and gene structure from wild progenitors to bread wheat

#### Information on the CDSs of bread wheat and its wild progenitors

High-confidence CDSs were obtained from public databases for bread wheat (Maccaferri et al., 2019), durum (Appels et al., 2018), wild emmer (Avni et al., 2017), *Ae. tauschii* (Luo et al., 2017), and rice (Sasaki et al., 2005). A total of 36 RNA-seq samples derived from 12 bread wheat tissues were collected (Appels et al., 2018), including seed, spike, stem, leaf, root, seedling, endosperm, inner pericarp, outer pericarp, stamen, pistillody stamen, and pistil. The CDSs of the expressed genes were extracted when TPM > 0.5. We defined genes showing tissue-specific expression as genes expressed in one bread wheat tissue but not expressed in the other 11 tissues.

#### Codon-composition

For each species, the frequencies of each of the 64 codon types were counted by reading all CDSs one by one, and the fractions of the 64 codons were merged into a vector to yield the codon composition. We conducted sliding window analysis using the in-house Python scripts with a sliding window size of 10 Mb and a step size of 5 Mb to determine the distribution of codons across the whole genome. The distribution of recombination was based on that from a previous study (Appels et al., 2018), and included 105 differential recombination zones on 21 bread wheat chromosomes. We calculated and compared the codon composition of five zones on each chromosome at the chromosomal window level, for which the significance of differences was evaluated using one-way analysis of variance (ANOVA) followed by Duncan’s Multiple Range Test (DMRT) as *post-hoc* correction tests, with a significance threshold of *p* < 0.05. Specifically, for ANOVA comparing codon composition among recombination zones (high, moderate, and low), we tested (64 codons × 21 chromosomes) 1,344 hypotheses per species per subgenome at the chromosome level, while for chromosome-window analyses, this number varied depending on the number of windows per chromosome.

Codon composition was calculated using in-house Python scripts at the level of the chromosome and chromosomal window for both expressed genes and genes showing tissue-specific expression. To examine the relationship between the expression level of genes and codon composition, we divided the expressed genes into five groups according to TPM: 0.5 to 1.5, 1.5 to 4.5, 4.5 to 13.5, 13.5 to 40.5, and more than 40.5; the codon composition was then calculated for each group. The resulting correlation coefficients (*r*-values) were consistently above 0.92 across all comparisons, indicating uniformly high levels of correlation.

#### Gene structure

According to reported annotation information for bread wheat and its wild progenitors, we calculated the GC3 of all transcripts per gene and the CDS length ratio of transcripts generated by each gene relative to all genes, number of transcripts per gene, the average CDS length of transcripts per gene, and TLC per gene using in-house Python scripts. CIHGP within bread wheat and its wild progenitors can be interactively browsed through the Triticeae-GeneTribe database (https://wheat.cau.edu.cn/TGT/). For the paired t-tests comparing codon proportions between wild progenitors and bread wheat (or between hybridization/doubling stages), we tested 64 codons per subgenome comparison, which corresponds to 64 hypotheses per comparison (AB subgenome and D subgenome analyzed separately. Meanwhile, for comparisons of GC contents (GC1, GC2, GC3, GC-CDS), 3–4 variables × 21 chromosomes = 63–84 hypotheses per comparison were evaluated.

### Testing the effect of hybridization and genome doubling on changes in codon composition from wild progenitors to bread wheat

#### Materials

In order to eliminate potential errors associated with genome annotation in public databases and accurately analyze changes of different genome transcripts during hybridization and genome doubling, we sequenced the full-length transcriptome of the plant materials using PacBio Iso-Seq and re-annotated the genome following the standard PacBio Iso-Seq pipeline. The specific information of the materials is as follows: four independent *Ae. tauschii* accessions were used as the male parent in the crosses, including CIae17: “2134”), CIae71: “M7-262”, PI511380: “KU-2126” and PI511382: “KU-2074” (referred to as P1, P2, P3, and P4, respectively). The female parent in each cross was ssp. *durum* cv. Langdon (L). Four triploid hybrids were produced (D1, D2, D3, and D4), as well as spontaneously doubled allohexaploid wheat (F1, F2, F3, and F4). Embryo rescue as the reported procedure was acquired for the production of triploid hybrids but not for the doubled allohexaploid wheat.

#### RNA sample preparation and transcriptome sequencing

RNA was extracted using an RNA prep Pure Plant kit (Tiangen, Beijing, China) from the youngest expanded leaf of pot-grown plants 15 days after vernalization. Three experimental replications were conducted for each of the four *Ae. tauschii* accessions, four triploid hybrids, four doubled wheat allohexaploids, and Langdon cultivar. A total of 0.5 μg of poly(A) RNA was reverse-transcribed into cDNA using the SMARTer™ PCR cDNA Synthesis Kit (TaKaRa). High-quality PCR products containing fragments 1–6 kb in length were purified to the optimal size. The SMRT sequencing library was constructed using 3.0-μg size-selected cDNA with the PacBio DNA Template Prep Kit 2.0 (Pacific Biosciences), and then sequenced on the PacBio RSII platform.

#### PacBio data analysis

The raw PacBio subreads were processed using the ccs program (https://github.com/nlhepler/pbccs) to produce consensus sequences. The full-length non-chimeric reads (FLNCs) were obtained by removing the sequencing primers using lima software (v2.2.0), and IsoSeq3 Refine (v3.4.0) was used to remove the inter-transcript linkers. The IsoSeq3 cluster algorithm was used to cluster FLNCs. The poly(A) tails and concatenated structures were removed, and similar sequences were clustered to obtain the high-quality consensus sequences.

#### Homeologous transcript assignment and filtering

The high-quality full-length iosforms were assigned to subgenomes based on their genomic alignment to the reference genome and sequence similarity to annotated sub genomic models. A transcript was assigned to a subgenome only when its best-supported genomic alignment corresponded uniquely to the subgenomic region. The mapping procedure used the high-qiality consensus sequences using Minimap2 with the parameters “-t 6 -ax splice --split-prefix -uf --secondary = no” (Li, 2018). Python script (collapse_isoforms_by_sam.py) from Cdna cupcake (https://github.com/Magdoll/cDNA_Cupcake) was used to remove redundant isoforms with the standard thresholds as minimum coverage 85%, minimum identity 90% (parameters: min-coverage = 0.85, min-identity = 0.9, and --dun-merge-5-shorter). TransDecoder v3.0.1 (http://transdecoder.sf.net) was used to predict open reading frames (ORFs) for all Iso-Seq isoforms, and the single best ORF was retained for each isoform (Haas et al., 2013). Homoeologous transcripts were distinguished according to their genomic locations and sequence identity. The isoforms with multiple competing genomic assignments or without sufficient evidence (as per selected threshold criteria) for a unique subgenomic assignment were excluded from downstream analyses. The retained transcripts were subsequently classified according to A, B, or D genomic locations, and their predicted coding sequences were used for codon composition analyses. The codon composition was calculated independently for each annotated subgenome to minimize the potential biases related to ambiguous transcript assignment.

## Data Availability

The original contributions presented in the study are included in the article/**Supplementary Material**. Further inquiries can be directed to the corresponding authors.
