# Supplementary material for: Association of hybridization and genome doubling with codon usage landscapes in the subgenomes of bread wheat
Source: Front Plant Sci. 2026 Sep 7;17:1857428. doi: 10.3389/fpls.2026.1857428 (PMC13590727; doi:10.3389/fpls.2026.1857428)
Supplement: Supplementary file 1 [file DataSheet1.zip › Figs Supplementary.docx]

**Association of Hybridization and genome doubling with codon usage landscapes in the subgenomes of bread wheat**

Xingchen Li^1*^, Mengyao Li^1*^,Yongchan Hao^2*^, Chengyu Liu^3^, Yuhang Gao^1^, Conghui Jiang^4^, Xueqiang Wang^5^, Muhammad Abdul Rehman Rashid^6#^, Yan Zhao^1#^

^1^ State Key Laboratory of Wheat Improvement, College of Agronomy, Shandong Agricultural University, Tai'an, Shandong, 271018, PR China.

^2^ State Key Laboratory of Wheat Improvement, Peking University Institute of Advanced Agricultural Sciences, Shandong Laboratory of Advanced Agricultural Sciences in Weifang, Shandong, 261325, PR China

^3^ College of Resources and Environment, Shandong Agricultural University, Tai’an 271018, China

^4^ Institute of Wetland Agriculture and Ecology, Shandong Academy of Agricultural Sciences, Jinan, Shandong 250100,PR China

^4^ Hainan Yazhou Bay Seed Laboratory, Sanya, Hainan, 572025, PR China.

^5^ Hainan Yazhou Bay Seed Laboratory, Sanya, Hainan, 572025, PR China.

^6^ Department of Bioinformatics and Biotechnology, Government College University Faisalabad, Faisalabad-38000, Pakistan

^*^ These authors contributed equally to this work.

^#^ Correspondence should be addressed to Yan Zhao (Email: yzhao1216@sdau.edu.cn; Tel: +86 538 8249278; Muhammad Abdul Rehman Rashid (Email: [marrashid@gcuf.edu.pk](mailto:marrashid@gcuf.edu.pk); Tel: +92 334 7915013).

**Fig. S1 Variation coefficient of each codon.** The codons are arranged by their variation coefficients in descending order.

**Fig. S2 Comparison of the proportion of each codon between the bread wheat AB and D subgenome at the chromosome level.** No significant difference was detected between the AB and D subgenome for each codon according to an independent-sample *t*-test.

**Fig. S3 Pairwise correlations in codon composition between pairs of bread wheat chromosomal widows.** For each 10-Mb sliding window, the proportions of 64 codons were integrated into a vector representing the codon composition of the sliding window, and the sliding step was 5 Mb. For each panel, correlations in codon composition are represented by the red and blue heat map at the bottom, and the dimensions of the differential recombination zones (R1, R2a, C, R2b, and R3) are indicated by the colored bar at the top.

**Fig. S4 Differences in codon composition between moderate- and low-recombination zones in bread wheat.** Colored boxes show significant differences in the proportions of codons, and gray boxes show the others. Orange indicates higher proportions in moderate-recombination zones than in low-recombination zones for the corresponding codon (27 codons with G or C in the third codon site and TTA), whereas blue indicates lower proportions in high-recombination zones than in low-recombination zones for the corresponding codon (31 codons with A or T at the third codon site, and TTG and CAG). TTA, TTG and CAG are marked by *.

**Fig. S5 Comparison of the proportion of each of 64 codons among high- (H), moderate- (M), and low-recombination (L) zones of bread wheat.** Significant differences in the proportions of codons were observed for 60, 61, and 43 codons between low- and high-recombination zones, low- and moderate-recombination zones, and high- and moderate-recombination zones, except for the codons marked by ×.

**Fig. S6 Pairwise correlations in codon composition between any two of the 21 bread wheat chromosomes based on differentially expressed genes.** Genes in each tissue were divided into five groups according to TPM: 0.5 to 1.5, 1.5 to 4.5, 4.5 to 13.5, 13.5 to 40.5, and more than 40.5. For each chromosome, the proportions of the 64 codons were integrated into a vector representing the codon composition of the chromosome. For each differential TPM group within each tissue, strong correlations in codon composition were observed among the 21 chromosomes with the mean *r* from 0.852 to 0.997.

**Fig. S7 Comparison of the proportion of each of the 64 codons between bread wheat and wild emmer in the AB subgenome (top) and between bread wheat and *Ae. tauschii* in the D subgenome (bottom).** Gray boxes indicate the proportions of codons within wild progenitors, whereas the other colors indicate the proportions of codons within bread wheat. Green and yellow indicate significant increases and decreases in the proportion from wild progenitors to bread wheat, respectively, and blue indicates no significant difference between bread wheat and its wild progenitor for the corresponding codon. Changes in the proportion of codons from wild progenitors to bread wheat are evident based on differences in the bases in the third codon position, with the exception of TTG.

**Fig. S8 Differences in the proportion of each of 64 codons between bread wheat and wild emmer in the AB subgenome (top) and between bread wheat and *Ae. tauschii* in the D subgenome (bottom).** Green and yellow indicate significant increases and decreases in the proportions of codons from wild progenitor to bread wheat, respectively, and blue indicates no significant difference between bread wheat and its wild progenitors in the corresponding codon. Changes in the proportions of codons from wild progenitors to bread wheat are evident based on differences in the bases in the third codon position, with the exception of TTG. The numbers above boxes (GCG and GAA) are extreme values of changes in proportions from progenitors to bread wheat in corresponding subgenome.

**Fig. S9 Comparison of the proportion of each of 64 codons between bread wheat and wild emmer using low-recombination zones of AB subgenome (top) and between bread wheat and *Ae. tauschii* using low-recombination zones of the D subgenome (bottom).** Gray boxes show the proportions of codons within wild progenitors, and the other colors indicate bread wheat. Green and yellow indicate significant increases and decreases in the proportions of codons from wild progenitors to bread wheat, respectively, and blue indicates no significant difference between bread wheat and its wild progenitor in the corresponding codon. Changes in the proportion of codons from wild progenitors to bread wheat were evident based on differences in the bases at the third codon position, with the exception of TTG.

**Fig. S10 Differences in the proportions of each of 64 codons between bread wheat and wild emmer using low-recombination zones of the AB subgenomes (top), and between bread wheat and *Ae. tauschii* using low-recombination zones of the D subgenome (bottom).** Green and yellow indicate significant increases and decreases in the proportion of codon from wild progenitors to bread wheat, respectively, and blue indicates no significant difference between bread wheat and its wild progenitors in the corresponding codon. Changes in the proportions of codons from wild progenitors to bread wheat were evident based on differences in the bases at the third codon position, with the exception of TTG. The numbers above boxes (GCG and GAA) are extreme values of changes in the proportions of codons from progenitors to bread wheat in corresponding subgenome.

**Fig. S11 GC-CDS (right) and GC-GS (left) comparison between bread wheat and its wild progenitors at the chromosomal window level.** Boxes show the GC-CDS and GC-GS across 10-Mb windows in the corresponding subgenome within bread wheat and its wild progenitors. The sliding step size was 5 Mb. Different letters above the boxes indicate significant differences (*p* < 0.05) according to Duncan’s test.

**Fig. S12 Distribution of GC-CDS and GC-GS along the chromosomes of bread wheat and its wild progenitors.** The GC-CDS and GC-GS of bread wheat and its wild progenitors are plotted on each chromosome for each 10-Mb sliding window. The sliding step size was 5 Mb. The GC-CDS of bread wheat, wild emmer, and *Ae. tauschii* is colored orange, green, and yellow, respectively. The GC-GS of bread wheat, wild emmer, and *Ae. tauschii* are colored gray because slight differences were observed between bread wheat and its wild progenitors. The five main differential recombination zones on each chromosome are separated by gray lines in each plot: R1 and R3 (distal regions), R2a and R2b (middle regions), and C (proximal region).

**Fig. S13 Increase in GC-CDS using all transcripts and transcripts of low-recombination zones at the chromosome level.** Different letters above the boxes indicate significant differences (*p* < 0.05) according to independent-sample *t*-tests.

**Fig. S14 Comparison of GC1, GC2, and GC3 between bread wheat and its wild progenitors.** Boxes show the GC content across 10-Mb windows in the corresponding subgenome within bread wheat and its wild progenitors. The sliding step size was 5 Mb. Different letters above the boxes indicate significant differences (*p* < 0.05) according to Duncan’s test.

**Fig. S15 Distribution of GC1 (top), GC2 (middle), and GC3 (bottom) along chromosomes of bread wheat and its wild progenitors.** For each 10-Mb sliding window, the GC content of bread wheat (orange), wild emmer (green), and *Ae. tauschii* (yellow) is plotted on corresponding chromosomes. The sliding step size was 5 Mb. The five main differential recombination zones on each chromosome are separated by gray lines in each plot: R1 and R3 (distal regions), R2a and R2b (middle regions), and C (proximal region).

**Fig. S16 Distribution of GC1, GC2, and GC3 along bread wheat chromosomes.** For each 10-Mb sliding window, GC1 (orange), GC2 (gray), and GC3 (green) of bread wheat are plotted on each chromosome. The sliding step size was 5 Mb. The five main differential recombination zones on each chromosome are separated by gray lines in each plot: R1 and R3 (distal regions), R2a and R2b (middle regions), and C (proximal region).

**Fig. S17** **Increases in GC1, GC2, and GC3 in the AB and D subgenome from wild progenitors to bread wheat at the chromosome level.** Different letters above the boxes indicate significant differences (*p* < 0.05) according to independent-sample *t*-tests.

**Fig. S18** Relationship between bread wheat GC-CDS and its local GC-GS at the chromosomal window level. GC-CDS and local GC-GS across 10-Mb sliding windows are plotted as GC-CDS versus GC-GS.

**Fig. S19** Relationship between increases in GC-CDS and changes in GC-GS from wild progenitors to bread wheat. Increases in GC-CDS and local GC-GS change across 10-Mb sliding windows were plotted as increase in GC-CDS versus change in GC-GS.

**Fig. S20 Comparison of the average number of transcripts per gene between bread wheat and its wild progenitors at the chromosome level.** Different letters above the boxes indicate significant differences (*p* < 0.05) according to Duncan’s test.

**Fig. S21** Correlation between GC3 of all transcripts per gene of wild progenitors and the relative changes in average CDS length of transcripts per gene from wild progenitors to bread wheat within its corresponding CIHGP in the AB (top) and D (bottom) subgenome.

**Fig. S22 Comparison of the average CDS length of transcripts per gene (codon number) between bread wheat and its wild progenitors at the chromosome level.** Different letters above the boxes indicate significant differences (*p* < 0.05) according to Duncan’s test.

**Fig. S23 GC3 of TCL and un-TLC in bread wheat and its wild progenitors at the gene (top) and chromosome (bottom) levels.** Different letters above the boxes indicate significant differences (*p* < 0.05) in bread wheat, wild emmer, and *Ae. tauschii* according to independent-sample *t*-tests.

**Fig. S24** **Comparison of the GC3 of transcripts with the longest CDS (TLC) per gene between bread wheat and its wild progenitors using CIHGPs at the gene (top) and chromosome (bottom) levels.** Different letters above boxes indicate significant differences (*p* < 0.05) according to paired *t*-tests.

**Fig. S25** **Increases in GC3 in the TLC of CIHGPs in AB and D subgenome from wild progenitors to bread wheat at the gene (left) and chromosome (right) levels.** Different letters above the boxes indicate significant differences (*p* < 0.05) according to independent-sample *t*-tests.

**Fig. S26 Proportions of three groups of CIHGPs in the AB and D subgenomes.**

**Fig. S27 Correlations in subgenomic codon composition in four triploid hybrids (T1 to T4, top) and four synthetic wheat allohexaploids (H1 to H4, bottom).**

**Fig. S28 Pairwise correlations in codon composition between any two chromosomal widows of four triploid hybrids (T1 to T4) and four doubled allohexaploids (H1 to H4).** For each 40-Mb sliding window, the proportions of 64 codons were integrated into a vector that represents the codon composition of the sliding window; the sliding step size was 20 Mb. Correlations in codon composition are indicated in red and blue, and the dimensions of the different recombination zones (R1, R2a, C, R2b, and R3) are indicated by the colored bar at the top.

**Fig. S29 Comparison of the proportions of each of 64 codons among one Langdon (L), four triploid hybrids (T), and four doubled allohexaploid wheat (H) in the AB subgenome.** Gray dots show the values of one Langdon, and colored violins show the values for four T and four H.

**Fig. S30 Comparison of the proportions of each of 64 codons among four *Ae. tauschii* (P), four triploid hybrids (T), and four doubled allohexaploid wheat (H) in the D subgenome.**

**Fig. S31 Changes in codon composition in low-recombination zones in bread wheat allopolyploidization, involving hybridization and genome doubling. a** Changes in the proportions of each codon in low-recombination zones of the AB subgenome from wild emmer to generated triploid hybrids (green) and from triploid hybrids to doubled allohexaploid wheat (red). Dark green and red boxes indicate significant changes in the proportions of codons according to the paired *t*-tests. The numbers above boxes are extreme values of changes in proportions during hybridization and genome doubling. **b** Changes in the proportions of each codon in low-recombination zones of the D subgenome from *Ae. tauschii* to triploid hybrids (green) and from triploid hybrids to doubled allohexaploid wheat (red).

**Fig. S32 GC-CDS, GC1, and GC2 of the AB and D subgenome within three generations.** Dots show the values for one Langdon, and boxes show the values from four *Ae. tauschii*, four triploid hybrids, and four doubled wheat allohexaploids. Different letters above boxes indicate significant differences (*p* < 0.05) according to Duncan’s test.

**Fig. S33** **Changes in GC3 and GC-CDS in the AB and D subgenome in bread wheat allopolyploidization.** Dots show the values for one wild emmer, and boxes show the values for four *Ae. tauschii*, four triploid hybrids, and four doubled wheat allohexaploids. Different letters above the boxes indicate significant differences (*p* < 0.05) according to paired *t*-tests.

**Fig. S34 GC-CDS, GC1, and GC2 of low-recombination zones in the AB and D subgenome within three generations.** Dots show the values for one wild emmer, and boxes show the values for four *Ae. tauschii*, four triploid hybrids, and four doubled wheat allohexaploids. Different letters above boxes indicate significant differences (*p* < 0.05) according to Duncan’s test.
